# Heparanase‐Loaded CAR T Extracellular Vesicles Remodel the Colorectal Tumour Microenvironment and Boost T Cell Antitumor Immunity

**DOI:** 10.1002/jev2.70310

**Published:** 2026-06-11

**Authors:** Songshan Zhu, Jun Yin, Weiqiang Yang, Xin Fu, Yiwei Zeng, Na Huang, Ling Zhang, Cong Yu, Ping Ouyang, Kaisong Huang, Rui Chen, Xiaolei Zhao, Dan Jiang, Guangxian Xu

**Affiliations:** ^1^ Guangdong Provincial Key Laboratory of Medical Immunology and Molecular Diagnostics, The First Dongguan Affiliated Hospital, School of Medical Technology Guangdong Medical University Dongguan People's Republic of China; ^2^ Dongguan Key Laboratory of Molecular Immunology and Cell Therapy Guangdong Medical University Dongguan People's Republic of China; ^3^ Institute of Transplant Medicine, The Second Affiliated Hospital of Guangxi Medical University, Guangxi Clinical Research Center for Organ Transplantation. Guangxi Key Laboratory of Organ Donation and Transplantation Nanning People's Republic of China; ^4^ Dongguan Key Laboratory of Medical Bioactive Molecular Developmental and Translational Research Guangdong Provincial Key Laboratory of Medical Immunology and Molecular Diagnostics Guangdong Medical University Dongguan People's Republic of China

**Keywords:** CAR T extracellular vesicles, heparanase, tumour microenvironment, extracellular matrix, colorectal cancer

## Abstract

Chimeric antigen receptor (CAR) T cell therapy has shown promise in solid tumours, but its efficacy is limited by dense extracellular matrix (ECM) that blocks T cell entry. We engineered mesothelin‐targeted CAR T cells to express heparanase (HPSE) fused to the truncated hepatitis A virus pX domain (pX‐Δ1‐30), enabling surface display of HPSE on extracellular vesicles for localized, pH‐dependent ECM degradation within the acidic tumour microenvironment while limiting systemic exposure. HPSE‐pX‐Δ1‐30 CAR T (referred to as HPSE CAR T) cells penetrated ECM mimics nearly fourfold more effectively than standard CAR T cells. They also expressed more TNF‐related apoptosis‐inducing ligand (TRAIL), Fas ligand (FasL), and perforin, leading to stronger tumour killing in 2D and 3D colorectal cancer models. HPSE‐pX‐Δ1‐30 CAR T–derived extracellular vesicles (EVs) retained CAR and chemokine receptors (CCR5/CCR7), carried apoptotic ligands, and were efficiently taken up by tumour cells and T cells. EV exposure promoted T cell proliferation, CCR5 expression, and central/stem‐like memory formation while lowering PD‐1 and CD57. In HCT116 xenografts, HPSE CAR T cells showed increased intratumoral infiltration, and EVs from these cells promoted infiltration of host T cells. Treatment reduced tumour burden, extended survival beyond 70 days, and did not cause systemic toxicity. These results highlight a dual strategy of ECM remodelling and immune modulation, offering a translational approach to overcome barriers to CAR T therapy in colorectal cancer.

## Graphical abstract

Dense ECM restricts CAR T efficacy in solid tumours. Zhu et al. design mesothelin‐targeted CAR T cells that secrete heparanase‐rich extracellular vesicles via a truncated hepatitis A virus pX domain (pX‐Δ1‐30), promoting ECM degradation, durable memory, and less exhausted phenotypes, leading to strong antitumor responses in colorectal cancer spheroids and tumour‐bearing mice.

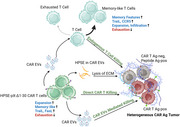

## 1 Introduction

Solid tumors often resist chimeric antigen receptor (CAR) T cell immunotherapy. Partly this resistance stems from a dense, stiff extracellular matrix (ECM) that limits T cell infiltration and reduces contact with tumour cells that express the target antigen (Hou et al., 2021). Among ECM components, heparan sulfate proteoglycan 2 (HSPG2) is particularly abundant in both malignant cells and tumour‐associated stroma (Johnson et al., 2024, Bassat et al., 2017), and its degradation by heparanase (HPSE) has been proposed to loosen this barrier and improve T cell access to the tumour core (Ostrovsky et al., 2007, Martinez et al., 2024, Caruana et al., 2015).

However, conventional CAR T manufacturing fails to sustain HPSE activity through the critical expansion and effector‐differentiation phases. We found that, although freshly isolated T cells upregulate HPSE transiently, enzyme levels sharply decline by the end of the second week—precisely when CAR T cells must proliferate and acquire cytotoxic function (Agliardi et al., 2025). At the same time, constitutive overexpression of HPSE carries the risk of off‐tumour ECM degradation, since HSPG2 is also expressed in essential organs such as lung, kidney, and vascular basement membranes. Thus, a strategy is needed to maintain ECM‐removing ability in the tumour while restricting enzyme action elsewhere. To this end, we engineered CAR T cells with HPSE fused to a truncated hepatitis A virus pX domain (pX‐Δ1‐30), which interacts with the ESCRT‐associated protein ALIX to target fused cargos into multivesicular bodies/extracellular vesicles (EVs) (Jiang et al., 2020, Bissig and Gruenberg, 2014). This design allows HPSE delivery via EV surface display rather than as a soluble free enzyme, thereby constraining its activity to the tumour microenvironment (TME) through contact‐dependent mechanisms.

In this study, we rigorously validate the pX‐Δ1‐30 platform for anchoring HPSE to EVs and characterize its impact on CAR T cell function. HPSE‐pX‐Δ1‐30 CAR T (referred to as HPSE CAR T) cells show improved ECM barrier penetration and more effective tumour lysis than standard CAR T in 2D and 3D models. Furthermore, these cells display enhanced memory‐like differentiation, reduced exhaustion marker expression, and a balanced cytokine profile—collectively offering a promising strategy to overcome ECM‐mediated resistance in solid tumors. HPSE‐pX‐Δ1‐30 CAR T–derived EVs (referred to as HPSE CAR T EVs) are readily taken up by HCT116 tumour cells and effectively target tumors *in vivo*, delivering cytotoxic signals via the TNF‐related apoptosis‐inducing ligand (TRAIL)–death receptor 5 (DR5) and Fas ligand (FasL)–Fas pathways to suppress tumour metabolism and proliferation, thereby promoting tumour cell death. These EVs also interact with T cells, are internalized, and modulate key functional attributes by altering chemokine receptor expression, apoptotic ligand profiles, and proliferative capacity, suggesting a dual role in tumour targeting and immune stimulation.

## 2 Materials and Methods

### 2.1 Cell Culture

Tumour cell lines HCT116, MKN45, MCF7, NCI‐H460, CAF, HEK293T and Jurkat T cells were obtained and authenticated from the Chinese Academy of Sciences. HCT116, MCF7, CAF and HEK293T cells were grown in high‐glucose Dulbecco's Modified Eagle Medium (DMEM) (Gibco, Cat. No. 10566016) containing 10% fetal bovine serum (FBS) (Gibco, Cat. No. 10091148) and 1% Penicillin/streptomycin (NCM, Cat. No. C100C5). MKN45, NCI‐H460 and Jurkat T cells were grown in Roswell Park Memorial Institute (RPMI) 1640 Medium (Gibco, Cat. No. 11875093) containing 10% FBS and 1% Penicillin/streptomycin. Cells were kept in 20% O_2_, 5% CO_2_ and at 37°C and passaged twice a week using 0.25% trypsin/EDTA (Gibco, Cat. no. 25200056). Cells were routinely negatively tested for mycoplasma.

### 2.2 Human Samples

For T cells, the healthy volunteers who provided blood were from our own group (samples were collected between June 2024 and August 2025). All donors provided informed written consent, and the use of human peripheral blood was approved by the Ethics Committee of The First Dongguan Affiliated Hospital of Guangdong Medical University.

### 2.3 Primary Human T Cell Isolation

Human peripheral blood mononuclear cells (PBMCs) were isolated from healthy donor buffy coats via density separation reagent (TBD, Cat. No. LTS1077) and density gradient centrifugation according to the manufacturer's protocol. Primary T cells were isolated from PBMCs via negative selection using the EasySep Human T Cell Iso Kit according to the manufacturer ’s instructions (STEMCELL Technologies, Cat. No. 17951). After stimulation with ImmunoCult HuCD3/CD28/CD2TCell Act (STEMCELL Technologies, Cat. No. 10990) for 24–72 h, activated T cells were randomly selected for lentivirus infection. T cells were cultured in complete human T cell medium consisting of X‐VIVO 15 medium (Lonza, Cat. No. 04–418Q) supplemented with 1% Penicillin/Streptomycin/Glutamine (100X) (Gibco, Cat. No. 15140122), 50 µmol/L β‐mercaptoethanol (Gibco, Cat. No. 21985023) and 10 ng/mL recombinant human IL‐2 (PeproTech, Cat. No. 200‐02‐50UG). Strategy for T Cell Source Selection: We employed a stratified validation strategy. Jurkat T cells were utilized as a genetically homogeneous model for the initial validation of construct expression and construct bioactivity, a strategy aligned with recent synthetic biology approaches and high‐throughput characterization protocols (Bloemberg et al., 2020). Primary human T cells isolated from healthy donor PBMCs were subsequently used for detailed mechanistic investigations and all *in vitro*/*in vivo* efficacy evaluations to ensure clinical relevance.

### 2.4 Immunofluorescence

Immunofluorescence analysis was performed as previously described (Caruana et al., 2015). Briefly, cells were fixed with 4% paraformaldehyde. After permeabilization with 0.1% Triton X‐100, cells were incubated with 5% goat serum (Solarbio, Cat. No. SL038) and 1% bovine serum albumin to block non‐specific binding. Cells were then stained with the primary Ab against human HPSE (Proteintech, Cat. No. 24529‐1‐AP) overnight. Cells were then probed with Alexa Fluor 555 goat anti‐rabbit secondary antibody for 1 h (Abcam, Cat. No. ab150078). 4',6‐diamidino‐2‐phenylindole (DAPI) (Biosharp, Cat. No. BL105A) was used as nuclear staining. Fluorescent signals were detected using a fluorescence microscope (Nexcope, NE910‐FL).

### 2.5 Spheroid Formation

The U‐shaped, 96‐well ultra‐low attachment (ULA) plates (S‐bio, cat. no. MS‐9384UZ) were seeded with a suspension of 180 µL cell culture media with 1 × 10^4^ cells per well. The culture media used was RPMI 1640, supplemented with 10% FBS, 1% Penicillin/streptomycin. The 96‐well ULA plates were sealed with Breathe‐Easy semipermeable tape (Diversified Biotech, cat. no. BEM‐1) to prevent evaporation. The spheroids were cultured at 37°C in an atmosphere of 5% CO_2_ under normoxia.

### 2.6 ATP Assay With CellTiter‐Glo 3D

Spheroids were cultured in 96‐well ULA plates for 7 days. Following co‐culture with T cells 3 days, medium was removed and cultures were washed in PBS twice to remove T cells. Individual spheroids were pipetted from PBS into white micro 96‐well plates (ThermoFisher, Cat. No. 236108) and an equal volume of CellTiter‐Glo 3D (cat. no. G9683, Promega) reagent was added. The contents were mixed for 5 min on an orbital shaker to induce cell lysis, while shielded from light. Luminescence readout (BioTek SYNERGY H1) was performed after 25 min incubation at 20°C (room temperature). Statistical significance between the groups was determined with a one‐way ANOVA and Tukey's multiple comparison test with significance level alpha 0.05.

### 2.7 Cell Viability Assay

HCT116 target cells were seeded into 96‐well plates at a density of 5 × 10^3^ cells per well. After 24 h of culture, effector cells (Jurkat T cell variants) were added to the target cells at the indicated effector‐to‐target (E:T) ratios in RPMI 1640 complete medium. The co‐culture system was incubated for 72 h. Subsequently, cell viability was determined using the Enhanced Cell Counting Kit‐8 (CCK‐8) (Elabscience, Cat. No. E‐CK‐A362) according to the manufacturer's instructions. Briefly, 10 µL of CCK‐8 solution was added to each well containing 100 µL of culture medium, followed by incubation at 37°C for 1–4 h. The absorbance (optical density, OD) was measured at 450 nm using a microplate reader.

### 2.8 RNA Isolation and qPCR

T cells, CAR T cells, and HPSE CAR T cells were collected and total RNA of the cells (2 × 10^6^ cells per group) was subsequently isolated via Ultrapure RNA Kit (CWBIO, Cat. No. CW0581S), and cDNA was obtained from total RNA via HiFiScript All‐in‐one RT Master Mix for qPCR (CWBIO, Cat. No. CW3371) according to the manufacturer's protocol.

### 2.9 Generation of Lentiviral Vectors and Transduction of T Lymphocytes

To test the function of pX‐Δ1‐30, the HPSE sequence was genetically fused with an N‐terminal 3×FLAG tag and a C‐terminal pX‐Δ1‐30 domain (arranged as 3×FLAG‐HPSE‐pX‐Δ1‐30). The standard CAR was designed as a second‐generation CAR comprising an anti‐ mesothelin (MSLN) scFv (Anetumab), a CD8 hinge and transmembrane domain, a 4‐1BB costimulatory domain, and a CD3ζ signalling domain. The HPSE CAR incorporated the same second‐generation CAR backbone linked to the HPSE–pX‐Δ1–30 construct via a 2A peptide.

Lentiviral particles encoding the indicated CAR constructs were commercially produced by GENEWIZ (Suzhou, China). To generate CAR T cells, T cells were transduced using a spinoculation protocol adapted from previously described methods (Pampusch et al., 2020). Briefly, primary T cells were activated for 24–72 h until significant blasting and cell clustering were observed. Activated T cells were resuspended in basal X‐VIVO 15 medium (lacking IL‐2 and antibiotics) and seeded in 96‐well plates at a density of 4 × 10^5^ cells per well in 100 µL. Lentiviral particles were added at a multiplicity of infection (MOI) of 8. Spinoculation was performed at 1000 × g for 10 min at 32°C. Plates were subsequently sealed and incubated at 37°C with 5% CO_2_. At 12 h post‐transduction, 100 µL of fresh basal X‐VIVO 15 medium was added to each well. At 24 h post‐transduction, cells were transferred to 24‐well plates containing 2 mL of complete human T cell medium (as defined above). During the 2‐week expansion phase, cell density was maintained at 1 × 10^6^ cells/mL by analysing viable cell counts and replenishing fresh complete medium every 2–3 days, strictly following standard T cell expansion protocols (STEMCELL Technologies). CAR expression was assessed by flow cytometry (FCM) three days after transduction.

### 2.10 Volume Calculations and 3D Rendering

Spheroid size (volume) was determined using AnaSP v2.0 (Piccinini et al., 2015, while 3D rendering of spheroid using ReViSP v2.3 (Piccinini et al., 2015, Piccinini et al., 2016).

### 2.11 Cytokine Production *in Vitro*

*In vitro*, 1.5 × 10^5^ CAR T cells were cocultured with a target tumour spheroid in 180 µL of medium without exogenous cytokines for 72 h in 96‐well ULA plates. The supernatant from each coculture was collected and analysed using a Six‐Cytokine Detection Kit (Flow Fluorescence Luminescence Method, WELLGROW, Cat. No. 281401HN) according to the manufacturer's instructions.

### 2.12 Matrigel Matrix Experimental and Infiltration Assessment of T/CAR T/HPSE CAR T Cells

Invasion experiment. A 24‐well plate (Falcon, No. 353504) with 8‐µm pore size filters (Falcon, No. 353097) was used to assess T cell infiltration. Diluted Matrigel was added to the upper side of the insert membrane and incubated at 37°C for 1 h to solidify, followed by a PBS wash. T cells were labelled with CellTracker red (ThermoFisher, Cat. No. C34565), resuspended in serum‐free medium at 5 × 10^5^ cells/mL, and 100 µL was added to the upper chamber. The lower chamber was filled with 650 µL RPMI 1640 complete medium with 20% FBS and hIL‐2. After 24 h, cells in the upper and lower chambers were imaged by fluorescence microscopy and counted with a hemocytometer.

EV‐induced T‐cell migration assay. The insert membranes were prepared as described above. T cells were labelled with red CellTracker (ThermoFisher, Cat. No. C34565), resuspended in serum‐free medium at 5 × 10^5^ cells/mL, and 100 µL and 25 µg HPSE CAR T EVs were added to the upper chamber. The lower chamber was filled with 650 µL RPMI 1640 complete medium with 20% FBS and hIL‐2. After 24 h, cells in the upper and lower chambers were imaged by fluorescence microscopy and counted with a hemocytometer.

### 2.13 EV Isolation and Characterization

CAR T cells and HPSE CAR T cells were incubated with anti‐G4S‐APC antibody (Hycells, clone B02H1, Cat. No. APS240612) in the dark for 1 h at 4°C. Samples were sorted on a Fusion Cell Sorter (Aurora CS, Cytek). The CAR T cells and HPSE CAR T cells were then cultured at a density of 2 × 10^6^ cells/ml for 48 h in complete human T cell medium consisting of X‐VIVO 15 medium supplemented with 1% Penicillin/Streptomycin/Glutamine, 50 µmol/L β‐mercaptoethanol and 10 ng/mL recombinant human IL‐2.

For EV purification from HEK293T cell culture supernatants, cells were cultured in media supplemented with 10% EV‐depleted FBS. Bovine EVs were depleted by 18 h centrifugation at 100,000 × g. Conditioned media were collected from cultures after 48–72 h. EVs were isolated by differential ultracentrifugation at 4°C (Thery et al., 2006, Peinado et al., 2012, Chen et al., 2018). Briefly, conditioned medium was first centrifuged at 300 × g for 5 min to pellet whole cells, 2000 × g for 10 min to remove dead cells, and 16,500 × g for 45 min to discard cell debris. Then supernatants were ultracentifugated at 100,000 × g for 2 h at 4°C (Beckman Coulter, Optima XPN‐100). Crucially, for mass‐balance fractionation assays, the supernatant remaining after the final 100,000 × g ultracentrifugation step was carefully collected and designated as the EV‐depleted supernatant. The pelleted EVs were suspended in PBS and collected by ultracentrifugation at 100,000 × g for 2 h. EVs were stored at −80°C prior to further analysis. In adherence to the MISEV2023 guidelines (Welsh et al., 2024), we refer to the vesicles isolated via ultracentrifugation collectively as EVs throughout this study.

For analysis by transmission electron microscopy, 10 µL of the EVs suspension was applied to a copper grid and allowed to adsorb for 5–10 min. Excess liquid was removed with filter paper. After blotting and air drying, the samples were stained with 2% uranyl acetate (10 µL) and imaged using an HT7700 transmission electron microscope (Hitachi, Japan). Nanoparticle tracking analysis (NanoSightTM) was performed as previously described (Welton et al., 2017). Briefly, the size distribution and particle concentration of the EVs were measured using the ZetaView Particle Metrix system (PMX‐120, Particle Metrix, Germany), while the zeta potential was assessed using size and zeta potential analysers (NanoBrook 90Plus, Brookhaven).

For FCM analysis, EVs were coupled to 4 µm aldehyde/sulfate latex beads by overnight incubation. The EV‐bound beads were then blocked with glycine to block residual binding sites and stained with the following fluorophore‐conjugated antibodies: CD63, CD9, CD81, 3×Flag, FasL, TRAIL, CAR, CD3, CD4, CD8, CD95, CCR7, CD45RA, CD27, PD‐1, CD28, CD57, HLA‐DR, CCR5. For the specific detection of HPSE, a two‐step indirect staining protocol was performed. EV‐bound beads were incubated with a primary antibody against HPSE, followed by incubation with an Alexa Fluor 555‐conjugated secondary antibody (Abcam, Cat. No. ab150078). Samples were detected using NovoCyte Opteon Spectral Flow Cytometer (Agilent) and analysed with FlowJo software (v10.8.1).

### 2.14 *In Vitro* T Cell Activation Assay

PBMCs were seeded into 48‐well plates at a density of 2 × 10^5^ cells per well in 500 µL of RPMI 1640 medium supplemented with 10% FBS. Notably, no exogenous IL‐2 was added to the culture medium to minimize non‐specific background activation. The cells were divided into three groups: 1. HPSE CAR T EVs group: Treated with 100 µg of HPSE CAR T EVs. 2. Positive Control: Treated with 2.5 µL of CD3/CD28/CD2 T cell activator. Negative Control: Treated with an equal volume of PBS. Cells were incubated at 37°C with 5% CO_2_ for 8, 24 and 48 h. At each time point, cells were harvested and washed with PBS. T cell activation markers were assessed using fluorochrome‐conjugated antibodies: anti‐CD3‐APC (UCHT1; Elabscience), anti‐CD25‐BV421 (M‐A251; BioLegend), and anti‐CD69‐PE‐Cy7 (FN50; BioLegend). Flow cytometric data were acquired using a NovoCyte Opteon Spectral Flow Cytometer (Agilent) and analysed using FlowJo software (v10.8.1).

Following the incubation of T cells (as described above), the culture supernatants were collected and centrifuged to remove cell debris. The levels of secreted IFN‐γ were quantified using the Human IFN gamma Uncoated ELISA Kit (Invitrogen; Thermo Fisher Scientific) according to the manufacturer's instructions. Briefly, 96‐well high‐affinity protein‐binding plates (Corning Costar 9018) were coated with capture antibody overnight at 4°C. After blocking and washing, samples and standards were added to the wells and incubated for 2 h at room temperature. The plates were then incubated with biotin‐conjugated detection antibody followed by Streptavidin‐HRP. Colour development was performed using TMB substrate, and the reaction was terminated with Stop Solution. The OD was measured at 450 nm using a microplate reader. All measurements were performed in triplicate.

### 2.15 Interaction of HPSE CAR T EVs With Recipient Cells and T Cell Proliferation

HPSE CAR T EVs were labelled with PKH26 (BestBio, cat. no. BB‐441125) following the manufacturer's instructions. After staining, EVs were washed twice with PBS by centrifugation at 10,000 × g for 10 min in a 100 kDa ultrafiltration tube (Merck millipore, cat. no. UFC5100BK) to remove excess dyes. To determine the cellular internalization of HPSE CAR T EVs by recipient cells, HCT116 cells (1 × 10^5^) or T cells (2 × 10^5^) were stained with 5 µM CellTracker Green CMFDA (ThermoFisher, cat. no. C2925) and incubated at 37°C for 40 min. Excess dye was removed and the cells were gently washed twice with PBS. PKH26‐labeled EVs were incubated with recipient cells. After 12 h, the incubated cells were fixed with 4% paraformaldehyde and stained with DAPI. Confocal images were obtained by Stellaris 5 (Leica). To evaluate the uptake efficiency of HPSE CAR T EVs, recipient cells were determined after 12 h exposure to PKH26‐labeled EVs using FCM.

For the proliferation assay, T cells were stained with 5 µM CellTracker Green CMFDA, according to the manufacturer's instruction, before incubation with the EVs. After 3 days in culture, T cell proliferation was determined by FCM.

### 2.16 Accumulation of HPSE CAR T EVs in Tumors

To determine whether HPSE CAR T EVs could target tumour site *in vivo*, they were labelled with 1,1'‐dioctadecyl‐3,3,3',3'‐tetramethylindotricarbocyanine iodide (DiR) following the manufacturer's instructions (Invitrogen, Cat. no. D12731). The labelled EVs were injected intraperitoneally into NOD scid gamma (NSG) mice with HCT116 subcutaneous tumors. The accumulation of DiR‐labelled EVs in tumour tissue was detected using an AniView100 Multi‐Model In Vivo Animal Imaging System after 3, 12 or 24 h (Biolight Biotechnology Co., Ltd).

### 2.17 Flow Cytometric Analysis

Surface staining of cells was performed using the following antibodies: anti‐CD3 (UCHT1), anti‐CD8 (SK1), anti‐CD4 (SK3), anti‐CD45RA (HI100), anti‐CD197 (2‐L1‐A), anti‐CD28 (CD28.2), anti‐CD27 (L128), anti‐CD95 (DX2), anti‐CD57 (NK‐1), anti‐PD‐1 (NAT105), anti‐CD195 (2D7), anti‐Flag (M2), anti‐G4S (B02H1), anti‐MSLN (EPR19025‐42). For the intracellular staining, cells were fixed, permeabilized, and stained with anti‐Perforin (B‐D48), anti‐TNF‐α (MAb11), anti‐GzmB (QA16A02), anti‐ IFN‐γ (4S.B3) as described before (Xiang et al., 2014). Details of the antibodies are listed in the Key Resources Table 1.

**TABLE 1:** Key resources table.

Reagent	Source	Cat. no
CD3	Servicebio	GB12014
Ki67	Servicebio	GB111499
HPSE	Proteintech	24529‐1‐AP
HSPG2	Abcam	ab2501
Collagen VI	Abcam	ab182744
Fibronectin	Abcam	ab2413
α‐SMA	Abcam	ab7817
CD146	Abcam	ab75769
PE Anti‐Mesothelin	Abcam	ab252136
HRP conjugated Goat Anti‐Mouse IgG	Servicebio	GB23301
HRP conjugated Goat Anti‐Rabbit IgG	Servicebio	GB23303
HRP conjugated Goat Anti‐Rat IgG	Servicebio	GB23302
APC Anti‐Human CD3	Elabscience	E‐AB‐F1230E
APC G4S	Hycells	APS240612
Hu HLA‐DR APC	BD	565128
Hu CD28 PE	BD	561793
Hu CD95 APC	BD	561978
Hu CCR7 (CD197) BV421	BD	566744
Hu CD27 BV605	BD	562656
Hu CD195 (CCR5) RB705	BD	570657
Hu CD45RA RB780	BD	569082
Hu CD4 BV786	BD	563877
Brilliant Stain Buffer Plus	BD	566385
Hu CD57 PE‐CF594	BD	562488
Hu CD3 BV510	BD	563109
Hu CD3 BV510	BD	563109

*Continued*		
Reagent	Source	Cat. no
Hu PD‐1 BV650	BD	564104
BV 421 anti‐human Fas‐L	BioLegend	306411
PE/Cyanine7 anti‐human CD81	BioLegend	349511
BV 711 anti‐human CD63	BioLegend	353041
PerCP/Cy5.5 anti‐human CD9	BioLegend	312109
BV 421 anti‐human CD25	BioLegend	356114
APC anti‐human Perforin	BioLegend	353311

### 2.18 Establishment and Treatment of HCT116 Tumors in NSG Mice

NSG mice were selected to enable stable engraftment of human colorectal tumors and to dissect the tumour‐intrinsic consequences of HPSE‐mediated ECM remodelling without confounding xenogeneic immune rejection. We acknowledge that conventional NSG mice lack endogenous human immune compartments. To specifically evaluate interactions between HPSE CAR T EVs and adaptive human immune cells, we incorporated a human T cell adoptive transfer model, allowing controlled assessment of bystander T cell recruitment and functional modulation *in vivo*. Fully humanized mouse models, which additionally recapitulate innate immune components, are discussed as an important future direction.

NSG mice were maintained in the Laboratory Animal Unit of the Guangdong Medical University. We designed a low effector‐to‐target (E:T) ratio model. A total of 5 × 10^5^ MSLN^+^ Fluc^+^ HCT116 cells were injected s.c. into each mouse. Seven days later, D‐luciferin was administered intraperitoneally to monitor tumour growth using an IVIS imaging system (IVIS Lumina Series III, PerkinElmer). Based on bioluminescence imaging, mice were randomly assigned to four groups. Each mouse then received 2.5 × 10^6^ T cells, CAR T cells, or HPSE CAR T cells via tail vein injection. The photon emission from tumour cells was expressed in photons (avg radiance (p/s/cm2/sr)). Local cytokines at the tumour site were assessed as described above. Additionally, the blood, spleen and major organs of the mice were carefully isolated for further analysis.

To evaluate the therapeutic effects of EVs on tumors, mice were injected via the tail vein with equal volumes of PBS or EVs (100 µg per mouse) every three days after tumour cell inoculation. Disease signs (disheveled hair, weight loss and reduced activity), tumour volume, and survival were monitored daily or measured at designated time points.

To examine the effect of EVs on T cell migration, T cells were stained by DiR following the manufacturer's instructions. Excess dye was removed by centrifugation, and 10 million labelled T cells were injected into each mouse. Mice subsequently received tail vein injections of equal volumes of PBS or EVs (100 µg per mouse) every four days.

### 2.19 Histological and Immunohistochemical Analysis

Mouse tissues were collected according to the study design. After sacrifice, the spleen, lung, liver, brain, kidney and heart were carefully dissected, dehydrated and hematoxylin and eosin (H&E) staining and immunohistochemistry (IHC). Details of the antibodies used for IHC are listed in the Key Resources Table 1.

### 2.20 Isolation and Flow Cytometric Analysis of Mouse Tumors, Spleens and Blood

Tumour tissues and spleens of mice were collected from different treatment groups for flow cytometric analysis. Tumour tissues were cut into 1–2 mm^3^ pieces and digested in 1 mg/mL collagenase IV (Biosharp, cat. no. BS165‐100 mg) and 0.1 mg/mL DNase I (MCE, cat. no. HY‐108882‐100 mg) for 30 min at 37°C. The cell suspension was applied to a cell strainer (100 µm, Biosharp, Cat. No. BS‐100‐CS‐N2), centrifuged at 300 × g for 5 min, and washed with cold PBS after removal of the supernatant and red blood cells.

### 2.21 Statistical Analysis

Data points were first excluded manually (for example, spheroids containing a dust particle when measuring dead signal, spheroids that were lost when pipetting to a white plate for measuring metabolic activity with CellTiter‐Glo 3D and so on). Afterwards, outliers were calculated via the ROUT method (*Q* = 1%) with GraphPad Prism v10, taking into account that the number of datapoints plotted in the graphs can be lower than N × n due to outlier exclusion. Statistical analysis was performed in GraphPad Prism v10. Unpaired comparisons were conducted using a one‐way ANOVA or Kruskal–Wallis test (after the mentioned transformations and the Shapiro–Wilk assessment for normality) with Tukey's or Dunn's multiple comparison test. Or unpaired Student's t‐test was used for different comparisons.

## 3 Results

### 3.1 PX‐Δ1–30–Mediated Anchoring of HPSE to EVs Enables Localized ECM Remodelling

**FIGURE 1:**
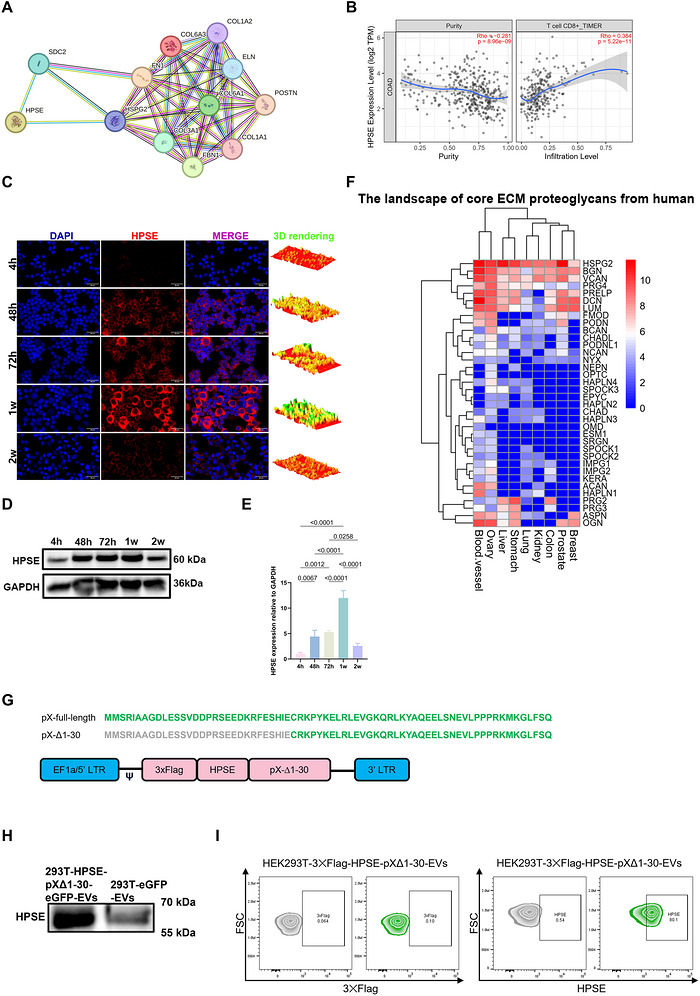
HPSE is dynamically regulated in T cells, and pX‐Δ1‐30 mediates its loading into EVs. (A) Protein‐protein interaction network depicting direct connections between HPSE and HSPG2, along with associated ECM components.(B) Scatter plots illustrating the correlation between HPSE expression and tumour purity (left), as well as CD8^+^ T cell infiltration levels (right) in colon adenocarcinoma (COAD). (C) Immunofluorescence images of HPSE expression (red) in freshly isolated T cells at indicated time points (4 h, 48 h, 72 h, 1 w, 2 w post‐isolation), with DAPI nuclear staining (blue) and merged channels; right panels show 3D renderings of HPSE distribution. Scale bar = 20 µm. (D) Western blot analysis of HPSE protein in T cells over time, with GAPDH as loading control. (E) Bar graph of qPCR results showing relative HPSE mRNA expression normalized to GAPDH across the different time points. *n* = 3 biological repeats, data are presented as mean ± SEM. Statistical significance between groups was determined by one‐way ANOVA followed by Tukey's multiple comparison test. (F) Heatmap of core ECM proteoglycan expression across human tissues, highlighting HSPG2 abundance in organs like liver, kidney, and colon. (G) Schematic representation of the engineered HPSE‐pX‐Δ1‐30 construct. Note: The lentiviral vector (GV513) utilizes a dual‐promoter system where gcGFP is expressed independently under the CBh promoter and is not genetically fused to the HPSE‐pX‐Δ1‐30 chimera. (H) Western blot analysis of HPSE in EVs derived from HPSE‐pX‐Δ1‐30‐HEK293T and eGFP‐HEK293T cells. (I) Flow cytometry analysis of surface marker expression on intact EVs. EVs derived from HEK293T cells transfected with the 3xFlag‐HPSE‐pX‐Δ1‐30 construct were coupled to aldehyde/sulfate latex beads and analysed. Left panel: Representative flow cytometry dot plots showing the expression of the N‐terminal 3xFlag tag compared to the Blank control. Right panel: Representative dot plots showing the expression of HPSE on the same EV population compared to the Blank control.

We examined gene expression data from nearly 30 cancer types and found that HSPG2, a major ECM component, is highly expressed both in tumour cells and in surrounding stromal cells (Figure S1A) (Han et al., 2023). A protein interaction network revealed a direct connection between HSPG2 and HPSE, suggesting HPSE may degrade HSPG2 to weaken the matrix barrier and facilitate T cell infiltration (Figure 1A–B; Figure S1B) (Cui et al., 2025).

To test whether additional HPSE is required in CAR T cells, we followed HPSE expression in freshly isolated T cells. Expression rose shortly after isolation, reaching a peak in week 1; we found that by week 2 HPSE expression had dropped by 70% (i.e. to approximately one third of peak level; *p* < 0.001), a critical period for CAR T expansion and effector differentiation (Figure 1C–E). This decline reveals a vulnerability in standard CAR T manufacturing that could limit tumour penetration. However, mass spectrometry analysis of ECM components (MatrisomeDB 2.0, https://matrisomedb.org/) (Shao et al., 2023) from various tissues confirmed that HSPG2 is highly expressed in several vital organs (Figure 1F; Figure S1C–D), underscoring a significant risk of off‐tumour activity when HPSE is constitutively overexpressed.

Given the off‐target concerns associated with widespread HPSE activity, we explored an EVs‐based strategy to restrict HPSE action to the TME. Given that CAR T‐derived EVs (CAR T EVs) preserve the CAR construct for specific antigen targeting (Fu et al., 2019) and T cell‐derived EVs (T EVs) have been shown to inherit parental tumour‐homing traits (Wang et al., 2020), we hypothesized that redirecting HPSE into these vesicles would allow localized ECM remodelling without systemic exposure. To implement this, we used a truncated pX domain from hepatitis A virus (pX‐Δ1‐30) (Figure 1G), known to promote protein incorporation into EVs via ALIX‐mediated sorting (Jiang et al., 2020).

We expressed HPSE fused to pX‐Δ1‐30 in HEK293T cells and found that this efficiently anchored HPSE to secreted EVs. Nanoparticle tracking, electron microscopy, and nanoscale FCM confirmed typical EV size, morphology, and expression of CD9, CD63, and CD81 (Figure S2A–E). Western blot analysis provided further evidence of HPSE incorporation (Figure 1H). To confirm that this specific EV incorporation relies on the pX‐Δ1‐30 sorting signal, we evaluated an HPSE‐only control. Although both constructs expressed equally in cell lysates (Figure S2F), HPSE lacking the pX tag was excluded from EVs (Figure S2G) and secreted solely as a soluble enzyme into the EV‐depleted supernatant (Figure S2H). In contrast, pX‐Δ1‐30 fusion abolished soluble secretion, selectively routing and anchoring HPSE to EVs alongside the ESCRT‐associated protein ALIX. Crucially, FCM characterization of intact EVs revealed a distinct membrane topology, where the N‐terminal FLAG tag was shielded within the EV lumen (surface‐negative), whereas the HPSE domain was robustly displayed on the EV surface (surface‐positive) (Figure 1I). This indicates efficient membrane anchoring of HPSE facilitated by the pX‐Δ1‐30 fusion, enabling potential direct access to extracellular substrates in the TME.

### 3.2 EV‐Directed Loading of HPSE Enhances CAR T Cell Penetration and Antitumor Activity

**FIGURE 2:**
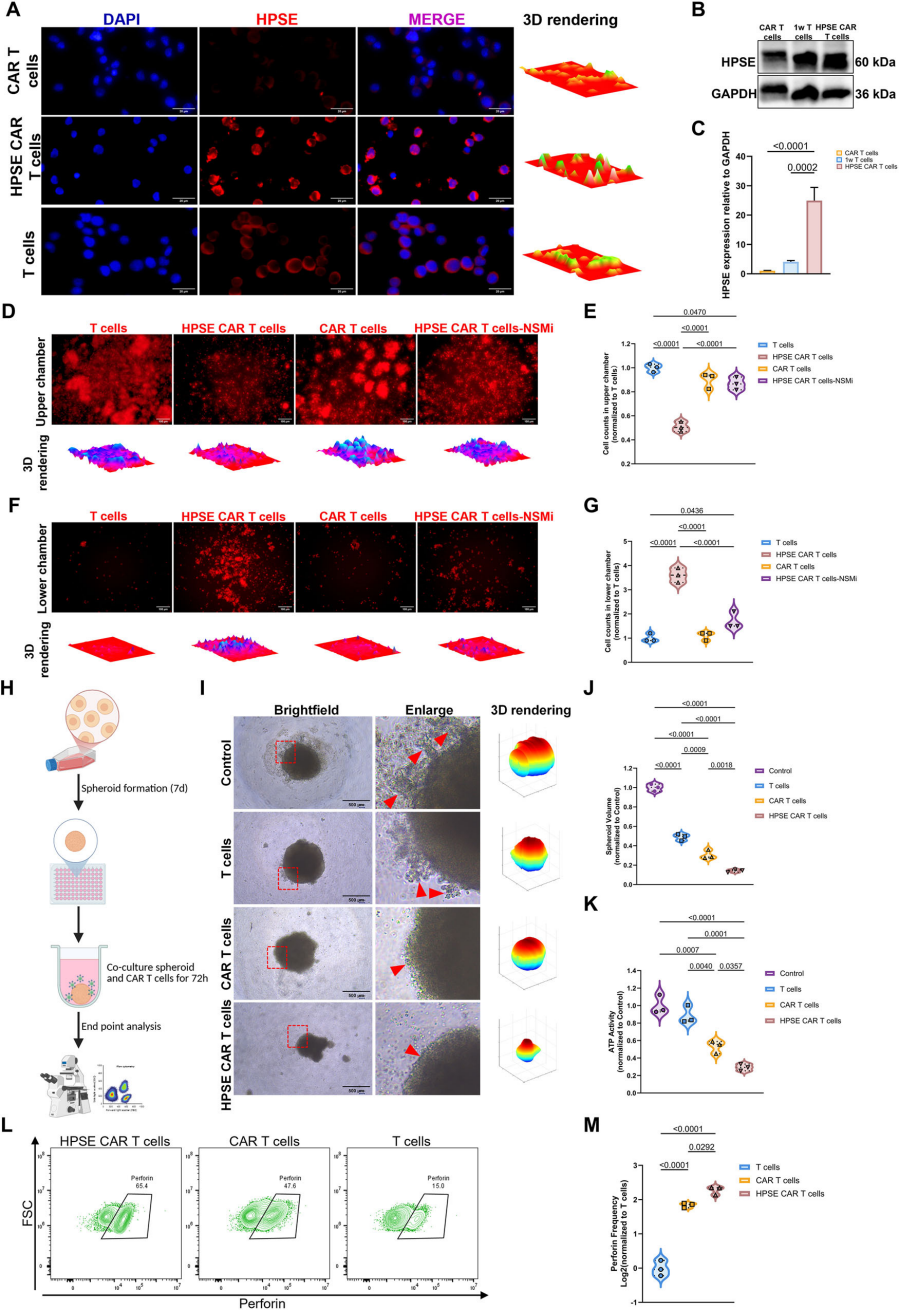
HPSE expression enhances CAR T cell penetration of matrix barriers and killing of tumour spheroids. (A) Immunofluorescence images showing HPSE (red) and DAPI (blue) staining in the indicated T cell groups, accompanied by 3D renderings of HPSE spatial distribution. Scale bar = 20 µm. (B) Western blot analysis of HPSE expression in 1‐week‐expanded T cells (1w T cells), CAR T cells, and HPSE CAR T cells, with GAPDH as a loading control; molecular weights indicated. (C) Bar graph detailing relative HPSE mRNA expression normalized to GAPDH across the indicated groups. (D, E) Fluorescence microscopy images of the upper chambers from a Matrigel Transwell assay (D) and corresponding quantification of retained cells (E). NSMi denotes treatment with a neutral sphingomyelinase inhibitor (GW4869) to block EV secretion. Scale bar = 100 µm. (F, G) Fluorescence images with 3D renderings of the lower Transwell chambers (F) and quantification of infiltrated cells that traversed the ECM‐mimicking barrier (G). (H) Schematic illustration showing the generation of HCT116 3D tumour spheroids and their co‐culture with T cells, CAR T cells or HPSE CAR T cells. (I) Brightfield images and 3D renderings of HCT116 spheroids after co‐culture with the indicated effector cells. Scale bar = 500 µm. (J–K) Quantification of spheroid volume and ATP‐based viability (CellTiter‐Glo 3D) from experiments in (I). (L) Flow cytometry showing intracellular perforin staining in T cells, CAR T cells and HPSE CAR T cells. (M) Quantification of perforin‐positive cells from the results in (L). (For C, E, G, J, K, and M, data are presented as the mean ± SEM of *n* = 3 biological replicates. Statistical significance was determined by one‐way ANOVA followed by Tukey's multiple comparison test.).

We engineered a MSLN‐targeted CAR that includes HPSE fused to pX‐Δ1‐30 (Figure S3A), aiming to sustain HPSE expression in CAR T cells and to deliver HPSE via EVs for localized ECM remodelling. Immunofluorescence showed that HPSE expression in HPSE CAR T cells remained high at two weeks, whereas standard CAR T cells and primary T cells exhibited a sharp decline (≈70% drop) by week 2 (*p* < 0.001) (Figure 2A–C).

HPSE CAR T cells exhibited strikingly enhanced invasive capacity, penetrating the ECM‐mimicking matrix nearly fourfold more effectively than standard CAR T cells. This robust infiltration was evident from the markedly higher numbers of HPSE CAR T cells observed in the lower chambers (Figure 2D–G). Importantly, pharmacologic blockade of EV secretion nearly abolished this advantage (Figure 2F–G), pinpointing EV‐delivered HPSE as a key driver of ECM penetration.

We examined MSLN across a panel of tumour cell lines, identified two with high expression, and selected HCT116 for further assays (Figure S3B). To rigorously verify the feasibility and functional potency of our genetic constructs before applying them to primary cells, we engineered Jurkat T cells with the HPSE‐CAR‐pX‐Δ1‐30 sequence. Utilizing Jurkat cells as a standardized screening model (Bloemberg et al., 2020), we sought to determine the construct's baseline bioactivity. In 2D co‐culture assays (E:T ratios of 10:1 and 3:1), HPSE CAR Jurkat T cells exhibited significantly enhanced lytic activity compared with conventional CAR Jurkat T cells (Figure S3C–D). We further extended this functional screening to 3D HCT116 spheroids to mimic the TME (Figure 2H), where HPSE CAR Jurkat cells again demonstrated superior killing capacity (Figure S3E–H). Having validated the construct's cytotoxic potential in this screening model, we proceeded to evaluate its therapeutic efficacy in primary human T cells (Vennin et al., 2023, Zheng et al., 2024).

To this end, we generated CAR T cells from primary T cells incorporating the HPSE overexpression module via the pX‐Δ1‐30 strategy (Figure S4A–C). Fractionation of HPSE CAR T supernatants revealed that HPSE is exclusively enriched in purified EVs and completely undetectable in the EV‐depleted fraction (Figure S4D), confirming the absolute absence of free soluble secretion. In spheroid assays, HPSE CAR T cells caused greater spheroid disruption and reduced cell viability more than standard CAR T cells (Figure 2I–K).

To explore potential mechanisms, we collected supernatants from co‐cultures and measured cytokines like IL‐2, IFN‐γ, TNF‐α, hIL‐4, hIL‐6, and hIL‐10. HPSE CAR T secreted much more IL‐2 than standard CAR T, but lower levels of IFN‐γ and TNF‐α (Figure S4E–F). That suggests stronger activation with less inflammatory risk.

We also looked at what molecules CAR T cells used to kill tumour cells. FCM showed TRAIL and FasL on HPSE CAR T surfaces were higher than on both primary T and standard CAR T cells (Figure S5A–D). Perforin was elevated too (Figure 2L–M), matching their better killing power. Importantly, HPSE CAR T cells displayed a favourable safety profile, with no observable off‐target toxicity or adverse effects in non‐tumour tissues or cells (Figure S5E–G).

### 3.3 HPSE CAR T Cells Exhibit Enhanced Memory Phenotypes and Lower Exhaustion Markers

**FIGURE 3:**
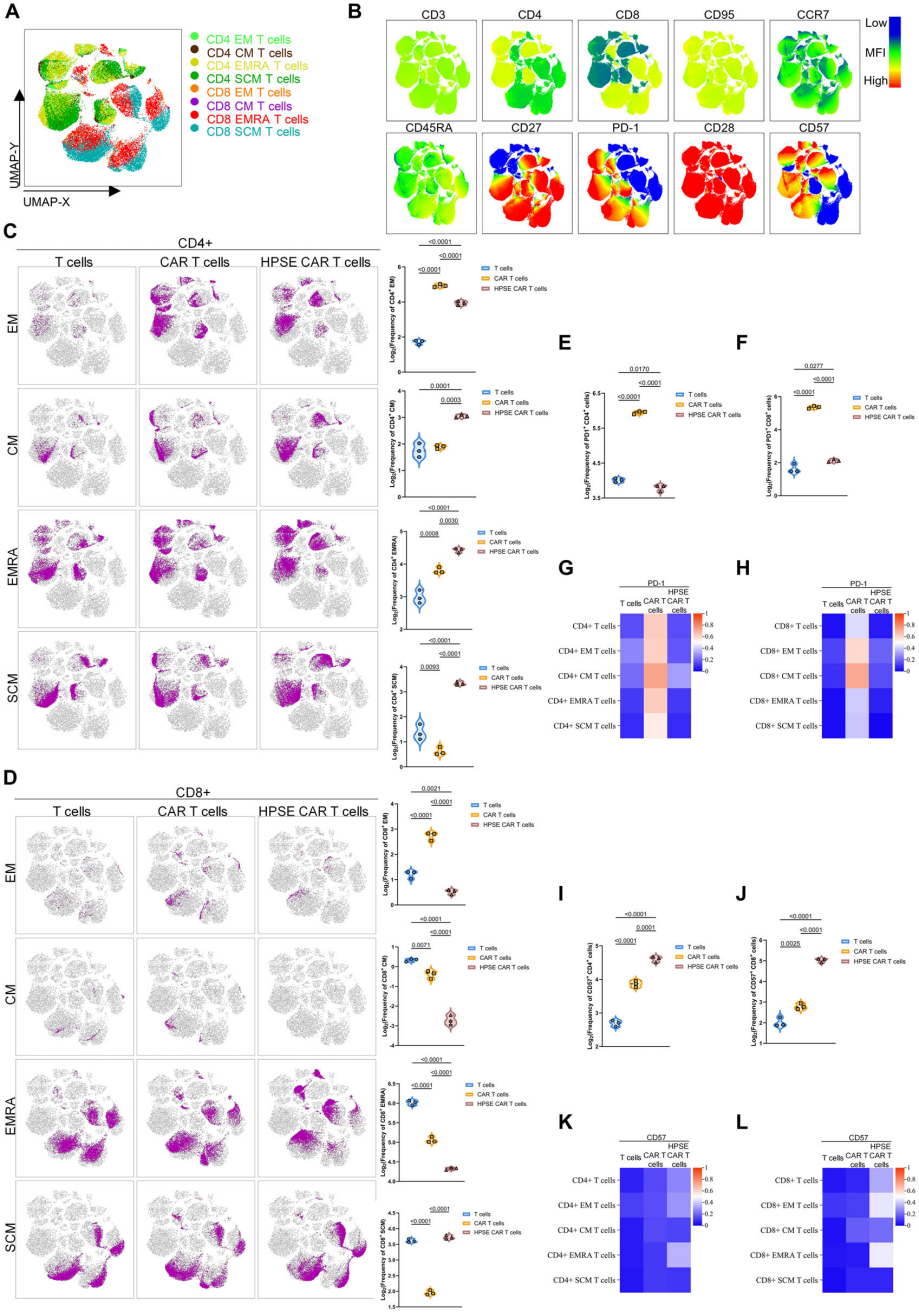
Phenotypic subsets and exhaustion marker expression in HPSE CAR T cells after co‐culture with tumor cells. (A) UMAP dimensional reduction analysis visualizing the clustered distribution of T cell memory subsets (EM, CM, EMRA, SCM) after co‐culture with HCT116 tumour cells. (B) UMAP plots of total T cells showing expression of the indicated markers. (C‐D) Memory subset distribution (SCM, CM, EM, EMRA) in CD4^+^ and CD8^+^ T cells across the three T cell groups, shown by UMAP plots and quantified by violin plots. (E‐F) Violin plots showing the frequency of PD‐1^+^ cells in CD4^+^ and CD8^+^ T cells across the T cell, CAR T, and HPSE CAR T groups. (G‐H) Heatmaps showing PD‐1 expression frequency across memory subsets of CD4^+^ and CD8^+^ T cells. (I‐J) Violin plots showing the frequency of CD57^+^ cells in CD4^+^ and CD8^+^ T cells across the T cell, CAR T, and HPSE CAR T groups. (K‐L) Heatmaps showing CD57 expression frequency across memory subsets of CD4^+^ and CD8^+^ T cells. (For C‐F and I‐J, *n* = 3 biological repeats, data are presented as mean ± SEM. Statistical significance between groups was determined by one‐way ANOVA followed by Tukey's multiple comparison test.).

To dissect phenotypic differences among HPSE CAR T cells, standard CAR T cells, and primary T cells after co‐culture with HCT116 spheroids, we applied Uniform Manifold Approximation and Projection (UMAP) for dimensionality reduction to visualize T cell subset distribution (Figure 3A–B; Figure S6A–C).

Notably, CD4^+^ HPSE CAR T cells displayed higher frequencies of central memory (CM) and stem cell memory (SCM) subsets compared to controls, indicating greater persistence potential. Similarly, CD8^+^ HPSE CAR T cells were enriched for SCM while showing fewer terminally differentiated cells (Figure 3C–D), consistent with a less exhausted, longer‐lived phenotype.

Given that T cell dysfunction in the TME comprises both functional exhaustion and replicative senescence, we assessed the classic exhaustion marker PD‐1 (Hui et al., 2017) and the terminal differentiation/senescence marker CD57 (Brenchley et al., 2003, Kared et al., 2016). Notably, the majority of HPSE CAR T subtypes exhibited lower expression of both PD‐1 (Figure 3E–H; Figure S6D–G) and CD57 (Figure 3I–L; Figure S6H–I) compared to standard CAR T cells and primary T cells, suggesting a more favourable exhaustion and senescence profile.

### 3.4 HPSE CAR T Cells Achieve Robust Tumour Control and Deep T Cell Infiltration *in Vivo*

**FIGURE 4:**
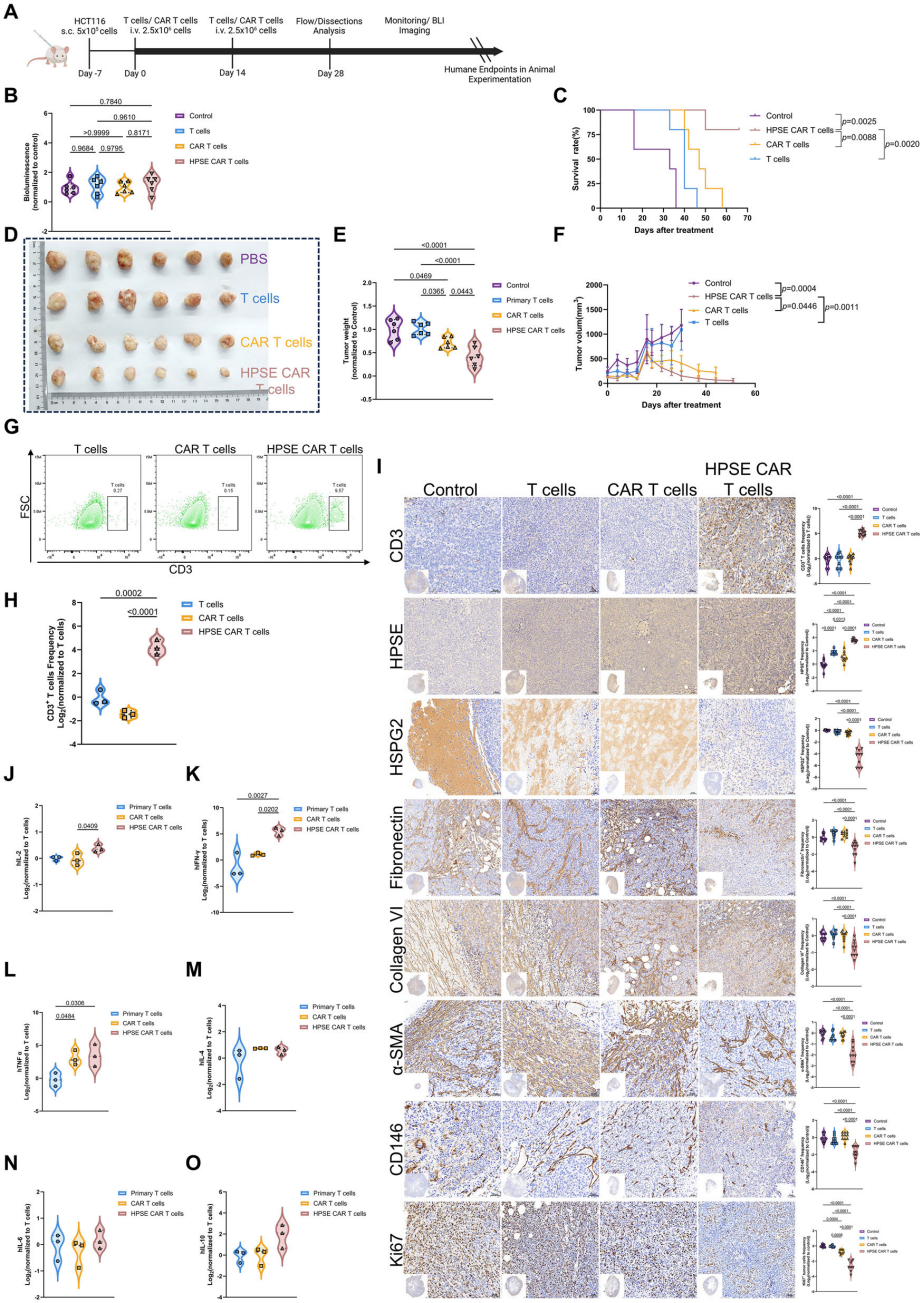
HPSE CAR T cells show in vivo antitumor activity and influence the tumor microenvironment in HCT116 xenografts. (A) Schematic of the experimental design and treatment timeline for the HCT116 xenograft model. (B) Quantification of tumour radiance from bioluminescence imaging before treatment (*n* = 6 per group). (C) Kaplan–Meier survival curves of NSG mice treated with PBS, T cells, CAR T cells, or HPSE CAR T cells (*n* = 5 per group). (D–F) Evaluation of tumour burden in split cohorts. (D) Representative images of excised tumors and (E) quantification of tumour weights from the dissection cohort (*n* = 6, sacrificed at Day 28). (F) Longitudinal tumour volume measurements of the survival cohort (*n* = 5, data from animals with complete longitudinal imaging records). Tumors shown in (D–E) were collected from a predefined dissection cohort sacrificed at Day 28 for mechanistic analysis, whereas data in (F) were derived from a separate cohort followed longitudinally for survival assessment. (G–H) Representative flow cytometry plots (G) and Log2‐normalized quantification (H) of human CD3^+^ T cell frequency in excised tumour tissues (*n* = 3 biological repeats). (I) Representative immunohistochemistry images and quantitative analysis of tumour sections showing T cell infiltration (CD3^+^), HPSE expression and ECM components (HPSE, HSPG2, Fibronectin, Collagen VI, α‐SMA), vascular integrity (CD146), and tumour cell proliferation (Ki‐67) across different treatment groups (*n* = 10 fields per sample). Scale bar = 50 µm. (J–O) Quantitative analysis showing the Log2‐normalized relative levels of the indicated cytokines in tumour lysates (*n* = 3 biological repeats). (Data are presented as mean ± SEM. For B, E, F, H, I, and J‐O, statistical significance between groups was determined by one‐way ANOVA followed by Tukey's multiple comparison test. In C, P values were determined by the log‐rank Mantel–Cox test.).

We evaluated HPSE CAR T efficacy in HCT116‐bearing NSG mice (Figure 4A). Baseline bioluminescence confirmed equivalent tumour burdens across groups prior to treatment (*p* > 0.05, Figure 4B), confirming equivalent disease status at the onset of therapy. This therapeutic impact translated into durable long‐term efficacy, as HPSE CAR T cells significantly extended median survival (> 70 vs. 30–45 days; *p* = 0.0020, Figure 4C). To further assess tumour control, a Day 28 dissection cohort revealed that HPSE CAR T treatment significantly reduced tumour weights compared to standard CAR T controls (*p* = 0.0443, Figure 4D–E). Accordingly, HPSE CAR T cells also robustly suppressed longitudinal tumour growth (*p* = 0.0446, Figure 4F; Figure S7A–B). Increased spleen indices further evidenced robust systemic T cell expansion (Figure S7C–D).

Enhanced efficacy correlated with improved T cell infiltration and proliferation at the tumour site (Wang et al., 2023). HPSE CAR T cells drove robust intratumoural expansion and functional activation of T cells. IHC and FCM revealed markedly increased CD3^+^ T cell infiltration in HPSE CAR T–treated tumors (Figure 4G–H). In IHC, CD3 staining revealed dense T cell accumulation in HPSE CAR T treated tumors, with approximately 32‐fold higher CD3^+^ cell density than other groups (Figure 4I). HPSE expression was strongly upregulated in HPSE CAR T–treated tumors, confirming local delivery via EVs. In contrast, the ECM scaffold component HSPG2 was markedly degraded in HPSE CAR T sections (≈70% reduction; Figure 4I). Fibronectin, collagen VI, and α‐SMA displayed similar fragmentation, indicating broad ECM remodelling (Figure 4I). CD146 staining, an indicator of endothelial integrity (Bardin et al., 2001), revealed irregular and disrupted vasculature in control, T cell and CAR T tumors. In contrast, HPSE CAR T treatment was associated with marked remodelling of tumour‐associated vasculature, characterized by fragmented and punctate CD146‐positive endothelial structures (Figure 4I). Ki‐67 staining was profoundly suppressed in HPSE CAR T tumors, consistent with reduced tumour cell proliferation and regression (Figure 4I).

Tumour cytokine profiling showed elevated IL‐2, IFN‐γ and TNF‐α with concomitant suppression of IL‐4, IL‐6 and IL‐10, indicating a Th1‐biased response without overt inflammation (Berger, 2000) (Figure 4J–O). Functionally, HPSE CAR T cells maintained high expression of apoptotic ligands (TRAIL, FasL) (Figure S7E–H) and cytotoxic mediators (granzyme B, TNF‐α, IFN‐γ) upon stimulation (Figure S7I–P), consistent with sustained effector capacity.

Systemic safety remained favourable. Serum cytokine analysis showed increased IL‐2 but lower IFN‐γ, TNF‐α, IL‐6, IL‐4 and IL‐10 relative to standard CAR T cells (Figure S7Q). Liver index and body weight were comparable across groups (Figure S8A–B), and H&E staining revealed no pathology in major organs, whereas tumour sections exhibited extensive necrosis (Figure S8C).

HPSE CAR T cells skewed splenic T cells toward long‐lived memory states. UMAP and FCM demonstrated enrichment of SCM subsets, with concomitant reduction of effector memory (EM) and terminally differentiated effector memory (EMRA) populations (Figure S9A–D). PD‐1 and CD57 expression was diminished, supporting reduced exhaustion and enhanced persistence (Figure S9E–G).

Because HPSE CAR T cells required EV release for their enhanced ECM invasion (Figure 2F–G), we asked whether secreted vesicles carry HPSE and other effectors that contribute to tumour targeting and killing. We therefore characterized EVs released by HPSE CAR T cells to define their molecular cargo and test their functional impact *in vitro* and *in vivo*.

### 3.5 HPSE CAR T EVs Home to Tumors and Induce Potent Cytotoxicity

Comprehensive FCM profiling of HPSE CAR T EVs revealed classical vesicle markers (CD9, CD63, CD81) alongside T cell and immunomodulatory markers (CD3, HLA‐DR, PD‐1, CD57) reflecting their parent cells (Figure S10A–D). Importantly, the EVs also express death ligands, including FasL and TRAIL (Figure S10D). Crucially, comparative analysis confirmed that both CAR T and HPSE CAR T EVs robustly retain surface CAR. Furthermore, while EVs from all activated groups displayed baseline trafficking and cytotoxic markers, HPSE CAR T EVs exhibited significantly enriched CCR5, CCR7, and these death ligands (FasL, TRAIL) (Figure S10E), uniquely empowering them with enhanced tumour homing and HPSE‐mediated matrix penetration.

**FIGURE 5:**
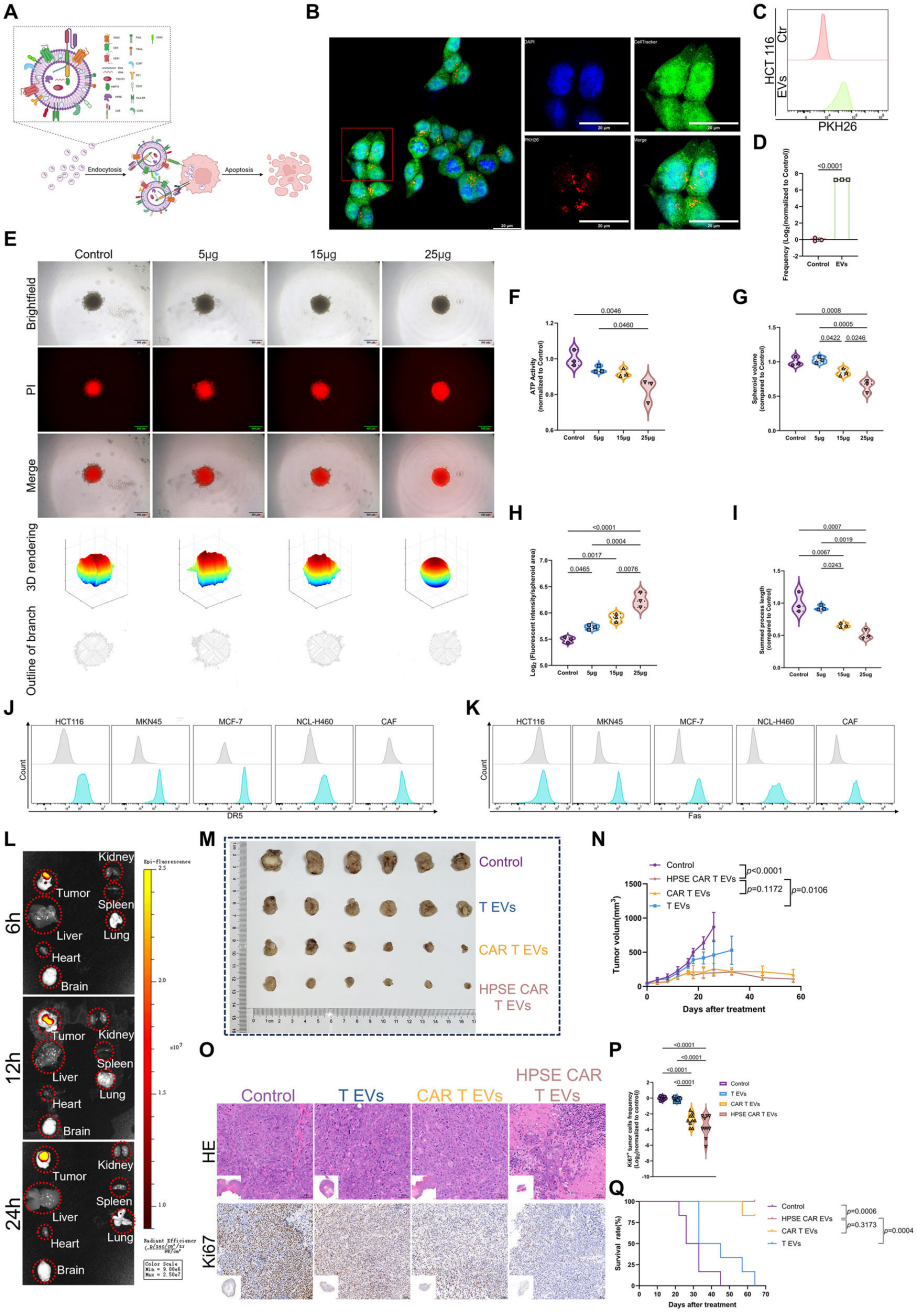
Tumour homing and antitumor activity of HPSE CAR T EVs *in vitro* and *in vivo*. (A) Schematic representation of the mechanism of HPSE CAR T EVs, illustrating their tumour cell internalization via endocytosis and the resulting activation of apoptotic pathways. (B) Confocal microscopy images demonstrating the efficient uptake of PKH26‐labeled EVs (red) by HCT116 cells (CellTracker Green for cytoplasm, DAPI for nuclei). Scale bar = 20 µm. (C, D) Flow cytometry histogram (C) and Log2‐normalized quantitative analysis (D) showing the specific cellular uptake of PKH26‐labeled HPSE CAR T EVs by HCT116 cells. (E) Representative images of HCT116 spheroids treated with increasing doses of EVs (0, 5, 15, and 25 µg) and stained with Propidium Iodide (PI, red) to visualize cell death. Bottom rows denote the corresponding 3D volumetric renderings and extracted branching outlines. Scale bar = 500 µm. (F–I) Violin plots quantifying relative ATP activity (F), spheroid volume (G), Log2‐normalized PI fluorescence intensity per spheroid area (H), and relative summed process length from 3D reconstructions (I) across the indicated EV doses. (J, K) Flow cytometry histograms showing the surface expression of the death receptors DR5 (J) and Fas (K) on a panel of tumour and stromal cell lines. (L) Ex vivo fluorescence imaging of major organs at 6, 12, and 24 h post‐injection of DiR‐labelled HPSE CAR T EVs, indicating preferential tumour accumulation. (M, N) Representative images of excised tumors (M) and longitudinal tumour growth curves (N) in HCT116 tumour‐bearing NSG mice following the indicated EV treatments. (O, P) Histological evaluation of excised tumour tissues. (O) Representative hematoxylin and eosin (H&E) and Ki67 staining images. The H&E staining in the HPSE CAR T EVs group reveals profound architectural remodelling and extensive coagulative necrosis (characterized by reduced viable cell density, eosinophilic debris, and nuclear pyknosis/karyorrhexis), contrasting with the dense viable architecture in controls. High‐magnification insets highlight specific necrotic features. (P) Quantitative analysis showing the Log2‐normalized frequency of Ki67^+^ tumour cells. (Q) Kaplan–Meier survival curves of HCT116 tumour‐bearing NSG mice treated with the indicated EV groups. (Data are presented as mean ± SEM. Statistical significance was determined by unpaired Student's t‐test for D (*n* = 3 biological repeats); one‐way ANOVA followed by Tukey's multiple comparison test for F–I (*n* = 3 biological repeats), N (*n* = 6 mice/group), and P (*n* = 10 fields/sample); and log‐rank Mantel–Cox test for Q (*n* = 5 mice/group).).

To explore the interaction between HPSE CAR T EVs and tumour cells, we first investigated their uptake mechanism in HCT116 cells. A schematic illustration (Figure 5A) depicts the proposed mode of action, where EVs are internalized by tumour cells via endocytosis, potentially triggering apoptosis. Confocal microscopy of PKH26‐labeled EVs (red), DAPI‐stained nuclei (blue), and CellTracker‐labelled cytoplasm (green) revealed robust intracellular red fluorescence, confirming efficient uptake by HCT116 cells (Figure 5B–D)

Having established their tumour cell internalization, we next assessed the cytotoxic activity of HPSE CAR T EVs using HCT116 spheroids. EV treatment (0, 5, 15, and 25 µg) resulted in a dose‐dependent increase in propidium iodide‐positive areas and progressive loss of spheroid architecture, with 3D reconstructions revealing marked structural collapse and reduced branching (Figure 5E–I). Mechanistically, the EV‐bound death ligands induce this profound cytotoxicity by triggering apoptosis in target cells via extrinsic signalling pathways (Figure 5J–K).

*In vivo* distribution studies provided additional insights into HPSE CAR T EVs functionality. Fluorescently labelled EVs (EV‐DiR) accumulated preferentially at tumour sites over time compared with the free DiR control (Figure 5L; Figure S10F–G), supporting their tumour‐homing capacity inferred from chemokine receptor expression. In NSG mice bearing HCT116 tumors, systemic administration of HPSE CAR T EVs significantly suppressed tumour growth compared to Control and T EVs groups. While exhibiting a consistent trend of enhancement over standard CAR T EVs (*p* = 0.1172, Figure 5M–N; Figure S11A–D), both engineered EVs demonstrated potent monotherapy efficacy. H&E staining revealed that HPSE CAR T EVs induced profound tumour destruction and architectural remodelling, characterized by extensive coagulative necrosis, nuclear pyknosis, and reduced viable cell density (Figure 5O). Conversely, normal organs showed no pathology (Figure S11E), and Ki‐67 staining confirmed suppressed tumour proliferation (Figure 5P). Survival was also prolonged (Figure 5Q), indicating strong antitumor efficacy with minimal systemic toxicity.

Together, these results demonstrate that HPSE CAR T EVs retain tumour‐targeting specificity, home to and penetrate tumors, and deliver pro‐apoptotic signals that induce robust, dose‐dependent tumour cell death.

### 3.6 HPSE CAR T EVs Modulate T Cell Memory and Exhaustion Phenotypes

**FIGURE 6:**
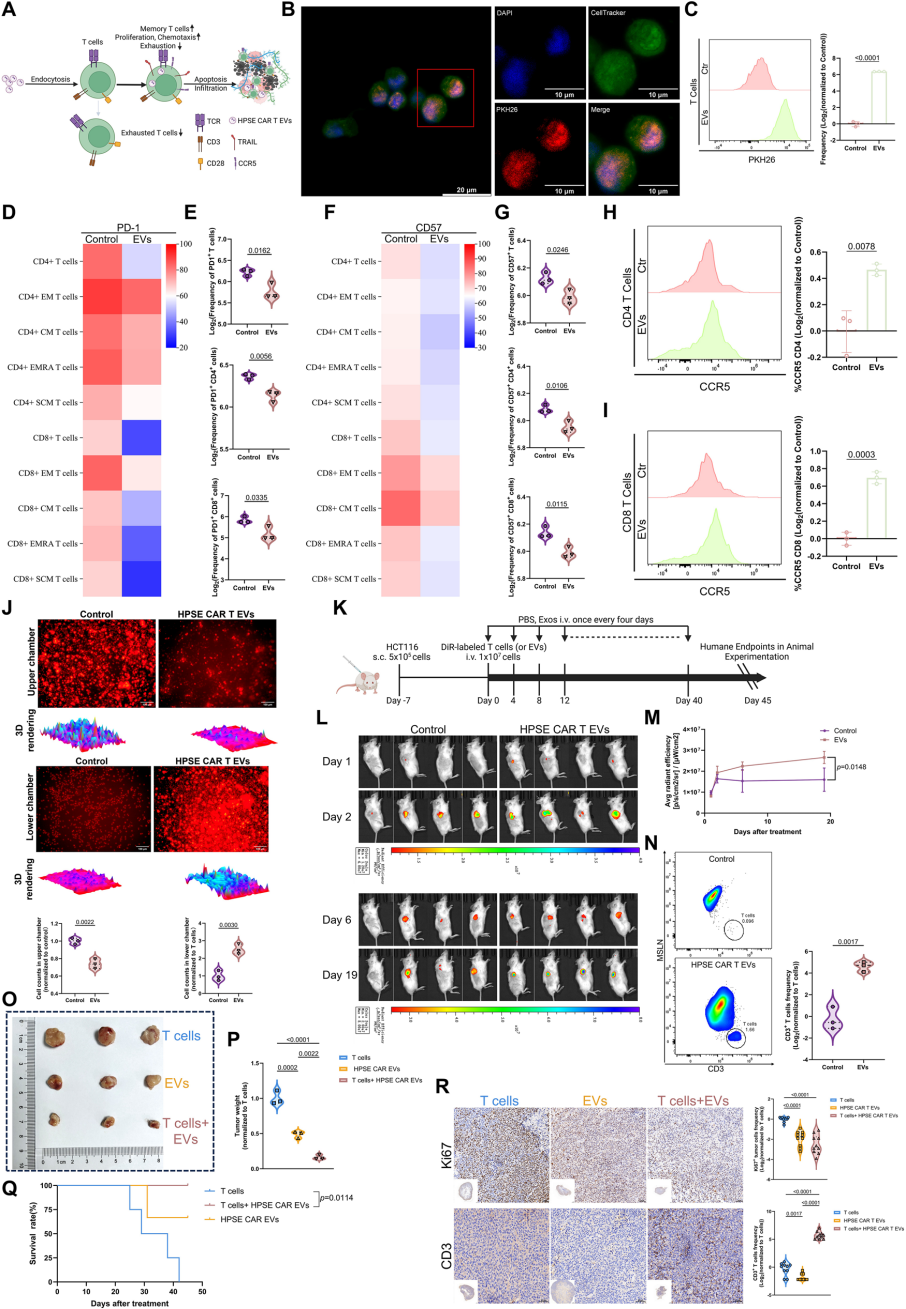
HPSE CAR T EVs enhance T cell function and antitumor immunity in the tumour microenvironment. (A) Schematic illustrating the proposed mechanisms by which HPSE CAR T EVs modulate the tumour microenvironment to promote T cell infiltration, enhance memory phenotypes, reduce exhaustion, and induce tumour cell apoptosis. (B) Confocal microscopy images demonstrating the efficient uptake of PKH26‐labeled EVs (red) by T cells (CellTracker Green for cytoplasm, DAPI for nuclei). Scale bars = 20 µm (left) and 10 µm (right). (C) Flow cytometry histogram (left) and Log2‐normalized quantitative analysis (right) showing the specific cellular uptake of PKH26‐labeled EVs by T cells. (D–G) Heatmaps depicting the relative expression frequency of PD‐1 (D) and CD57 (F) across memory subsets of CD4^+^ and CD8^+^ T cells. Corresponding violin plots quantify the Log2‐transformed frequencies of PD‐1^+^ (E) and CD57^+^ (G) cells across the indicated T cell populations. (H, I) Flow cytometry histograms and corresponding Log2‐normalized quantification of CCR5 surface expression on CD4^+^ (H) and CD8^+^ (I) T cells following EV treatment. (J) Fluorescence microscopy images of the upper and lower Transwell chambers, accompanied by 3D renderings and corresponding quantification of cell infiltration. Scale bar = 100 µm. (K) Schematic of the *in vivo* HCT116 tumour model, detailing the timing of subcutaneous tumour inoculation, DiR‐labelled T cell injection, and repeated intravenous EV or PBS administrations. (L, M) *In vivo* fluorescence imaging at Days 1, 2, 6, and 19 (L) and corresponding quantitative tracking of average radiant efficiency (M), illustrating the enhanced accumulation of co‐injected DiR‐labelled T cells at the tumour site following EV treatment. (N) Flow cytometry scatter plots and Log2‐normalized quantification (violin plots) of the frequency of infiltrating CD3^+^ T cells isolated from excised tumour tissues. (O, P) Representative images of excised tumors (O) and relative quantification of tumour weights (P) at the study endpoint. (Q) Kaplan–Meier survival curves of HCT116 tumour‐bearing NSG mice across the indicated treatment groups. (R) Representative immunohistochemistry images and Log2‐normalized quantitative analysis of tumour sections evaluating tumour cell proliferation (Ki67) and T cell infiltration (CD3) across treatment groups. Scale bar = 50 µm. (Data are presented as mean ± SEM. Statistical significance was determined by unpaired Student's *t*‐test for two‐group comparisons in C, E, G, H, I, J, M, and N; one‐way ANOVA followed by Tukey's multiple comparison test for multi‐group comparisons in P and R; and log‐rank Mantel–Cox test for survival analysis in Q. *n* = 3 biological replicates for *in vitro* assays unless otherwise noted; for *in vivo* assays, *n* = 4 mice/group for L‐M, *n* = 3 mice/group for O‐P, *n* ≥ 3 mice/group for Q, and *n* = 10 fields/sample for R.).

To determine whether HPSE CAR T EVs modulate T cell function, we first assessed their uptake by T cells. Confocal imaging revealed robust co‐localization of PKH26‐labeled EVs (red) with CellTracker‐labelled cytoplasm (green) and DAPI‐stained nuclei (blue), confirming efficient EV internalization (Figure 6A–B). Quantitative fluorescence analysis showed a significant increase in EV signal intensity compared with controls (*p* < 0.0001, Figure 6C), and FCM analysis further validated their uptake. This highly efficient internalization by both tumour and T cells identifies these EVs as effective cargo delivery vehicles, supporting subsequent T‐cell functional modulation and synergistic cytotoxicity.

Next, we evaluated how HPSE CAR T EVs influence T cell phenotypes (Figure S12A–B). EV treatment led to a marked enrichment of CM and SCM subsets within both CD4^+^ and CD8^+^ populations (Figure S12C–D), indicating a shift toward a less differentiated, longer‐lived T cell phenotype. In parallel, expression of the exhaustion marker PD‐1 and the senescence marker CD57 was significantly reduced across these subsets (Figure 6D–G; Figure S12E–F), supporting enhanced persistence and sustained proliferative capacity. Notably, exposure to HPSE CAR T EVs significantly increased the CD4/CD8 ratio (Figure S12G; *p* < 0.0001), reflecting preferential enrichment of CD4^+^ T cells. This shift is biologically meaningful. CD4^+^ T cells provide critical help that confers a cytotoxic effector program to CD8^+^ T cells, which includes downregulation of coinhibitory receptors and enhanced tissue invasiveness. Additionally, CD4^+^ T cells also directly contribute to cytotoxicity through extrinsic apoptotic pathways, as evidenced by upregulated TRAIL expression observed in EV‐treated T cells (Figure S12H) (Castilho et al., 2022, Ahrends et al., 2017, Borst et al., 2018). Such CD4^+^‐skewed responses have been linked to effective antitumor immunity in cancer immunotherapy contexts (Borst et al., 2018). Functionally, HPSE CAR T EVs significantly upregulated TRAIL (Figure S12H) while maintaining FasL levels (Figure S12I) on T cells, thereby amplifying cytotoxicity via extrinsic apoptotic pathways alongside the EVs’ intrinsic death ligands.

HPSE CAR T EVs also significantly enhanced T cell proliferation *in vitro*, as indicated by elevated proliferation marker expression and pronounced peak shifts in FCM, with quantitative analysis showing higher division rates (Figure S12J–M). Consistent with these findings, mice that received T cells plus EV treatment showed increased spleen weight and spleen index (Figure S13A–B). Moreover, exposure of primary T cells to HPSE CAR T EVs *in vitro* led to upregulation of CCR5 on both CD4^+^ and CD8^+^ subsets (Figure 6H–I) and enhanced migration in transwell assays (Figure 6J). In NSG mice bearing HCT116 tumors, DiR‐labelled T cells followed by EV treatment showed significantly greater tumour accumulation (Figure 6K–M) and increased frequency of human CD3^+^ T cells by FCM (*p* = 0.0017; Figure 6N).

Building on our *in vitro* data that HPSE CAR T EVs increase T cell proliferation, CCR5 expression, and migratory capacity, we next evaluated their antitumor efficacy and safety *in vivo*. In NSG mice bearing HCT116 tumors, treatment with HPSE CAR T EVs—given alone or together with primary T cells—markedly suppressed tumour growth by bioluminescence imaging and tumour volume measurements (Figure S13C–F). Endpoint tumour weights were lowest in the group receiving T cells plus HPSE CAR T EVs compared with T cells or EVs alone (Figure 6O–P), and survival was significantly prolonged in the EV‐treated cohorts (*p* = 0.0114; Figure 6Q). Importantly, the combination treatments were well tolerated; mice maintained stable body weights (Figure S13G) and showed no signs of hepatomegaly, with comparable liver indices across all groups (Figure S13H). We then profiled splenic T cells from treated mice. Mice receiving HPSE CAR T EVs showed higher frequencies of CM and SCM cells in both CD4^+^ and CD8^+^ compartments. These changes were accompanied by lower expression of PD‐1 and CD57 on splenic T cells, indicating a shift toward less exhausted phenotypes and diminished replicative senescence (Figure S13I–L). Finally, splenic T cells from EV‐treated mice displayed increased CCR5, FasL, and TRAIL expression, a profile consistent with enhanced trafficking capacity and augmented death‐ligand potential (Figure S13M–O).

H&E analysis revealed markedly increased CD3^+^ T cell infiltration in tumour tissues from mice treated with T cells plus HPSE CAR T EVs, whereas Ki‐67 staining was lowest in the same group, suggesting reduced tumour cell proliferation (Figure 6R). HPSE CAR T cells and their derived EVs exhibited a favourable safety profile, with no evidence of graft‐versus‐host disease (GVHD)‐like pathology. In the humanized NSG mouse model, treatment did not induce hepatomegaly, as indicated by comparable liver indices across all experimental groups (Figure S8A; Figure S13H). In addition, mice maintained stable body weight throughout the treatment course, with no signs of rapid cachexia typically associated with systemic alloreactivity (Figure S8B; Figure S11E; Figure S13G). H&E staining of major organs, including classical GVHD target tissues such as liver, lung, and kidney, revealed preserved tissue architecture without detectable lymphocytic infiltration, in sharp contrast to the extensive necrosis observed within tumour tissues (Figure S8C). Consistently, serum cytokine profiling demonstrated a controlled immune response without features of a systemic cytokine storm (Figure S7Q).

To further mechanistically assess the risk of systemic immunotoxicity, we examined whether HPSE CAR T EVs could induce non‐specific T cell activation in the absence of tumour antigen. Following established protocols for evaluating the immunogenicity of engineered EVs (Shi et al., 2020), resting human PBMCs were incubated with EVs for up to 48 h. Flow cytometric analysis revealed no significant upregulation of the early activation marker CD69 or the late activation marker CD25 compared with PBS‐treated controls (Figure S13P–Q). Consistently, IFN‐γ secretion remained at baseline levels (Figure S13R), whereas robust activation was observed in the CD3/CD28/CD2 agonist–treated positive control. These findings indicate that HPSE CAR T EVs are immunologically inert in the absence of MSLN‐positive tumour cells, supporting their antigen‐dependent activity and minimal risk of off‐target GVHD‐like responses.

## 4 Discussion

The HPSE‐pX‐Δ1‐30 CAR T construct represents a unique modular platform that marries potent matrix‐degrading activity with built‐in safety controls. Unlike conventional CAR T cells that lose HPSE during expansion, our design re‐introduces an HPSE domain fused to a pX‐Δ1‐30. The pX C‐terminus is known to bind the vesicular sorting factor ALIX, efficiently loading fused proteins into vesicles (Jiang et al., 2020, Martin and Lemon, 2006). In practice, this means that HPSE is packaged into secreted EVs, rather than being freely secreted. As others have shown, CAR T cells engineered to express HPSE degrade heparan sulfate proteoglycans and markedly enhance tumour infiltration and antitumor efficacy (Caruana et al., 2015). Our modular fusion likely preserves the enzymatic core of HPSE while tethering it to multivesicular bodies via pX‐Δ1‐30, thus controlling its activity and confining ECM digestion to the TME. In effect, the CAR T cells gain the ability to carve channels through the dense stroma, releasing bound chemokines that further attract T cells (Caruana et al., 2015). By contrast, the absence of free HPSE in circulation minimizes the risk of off‐tumour matrix damage. In sum, the combination of HPSE with the pX‐Δ1‐30 EV‐targeting motif provides enhanced cytotoxicity (through superior tissue penetration) together with a built‐in safety switch that limits HPSE dispersion.

Mechanistically, FCM of intact EVs revealed a distinct ‘Dual‐Anchored’ topology: EVs were surface‐positive for HPSE yet surface‐negative for the N‐terminal Flag tag (Figure 1I). This suggests that while pX drives luminal loading, a functional fraction of HPSE is retained on the surface (consistent with anchoring via proteoglycans (Bandari et al., 2018)) enabling direct, contact‐dependent ECM degradation (Goldshmidt et al., 2002). Importantly, bioactivity is spatially restricted by the enzyme's intrinsic pH dependence (inactive at physiological pH 7.4; active at intratumoral pH 5.0–6.0) (Vlodavsky et al., 1999). This localized potency was confirmed *in vivo* by the specific depletion of the HPSE substrate HSPG2 within tumour tissues (Figure 4I), validating safe, tumour‐confined matrix remodelling.

Functionally, HPSE CAR T cells exhibited superior 2D and 3D cytotoxicity, engaged a dual cytotoxic program via upregulated TRAIL/FasL/perforin, and produced a safer cytokine profile (high IL‐2, low IFN‐γ/TNF‐α) with reduced risk of cytokine release syndrome. They also maintained greater CM and SCM phenotypes. Importantly, they displayed lower levels of PD‐1, indicating a less exhausted state, and lower levels of CD57, a marker of replicative senescence associated with proliferation arrest. One potential mechanism underlying this phenotype is that ECM degradation by HPSE alleviates the physical and metabolic constraints of the TME, improving oxygen and nutrient diffusion. Dense ECM is known to create hypoxic, nutrient‐deprived niches that drive T cell dysfunction and accelerate differentiation toward a senescent (CD57^+^) phenotype (Jayaprakash et al., 2022). By remodelling the ECM, HPSE may restore a more permissive milieu that favours the maintenance of T cells in a less differentiated, non‐senescent state. This aligns with prior observations that matrix stiffness and hypoxia synergize to impair T cell fitness, while stromal remodelling can rejuvenate their metabolic and functional states (Scharping et al., 2021).

Remarkably, the HPSE‐ pX‐Δ1‐30‐CAR T system yields an independent therapeutic entity: HPSE CAR T EVs. These nanoscale vesicles inherit both the antigen specificity and killing machinery of their parent cells. Indeed, recent studies have demonstrated that EVs released by activated CAR T cells carry the CAR on their surface and high levels of cytotoxic granules, and can mediate potent tumour cell lysis (Fu et al., 2019, Wang et al., 2020, Sani et al., 2024). Critically, cell‐free EVs can be dosed like a biologic drug: they circulate through blood, passively accumulate in tumors via the enhanced permeability effect, and even traverse tight barriers (e.g. the blood–brain barrier) (Syn et al., 2017, Robbins and Morelli, 2014). CAR T EVs could replace CAR T cells as the primary effector units, providing a clinically controllable, cell‐free platform that responds to tumour complexity (Pitt et al., 2016, Shah et al., 2015). Consistent with this concept, HPSE CAR T EVs directly kill antigen‐positive tumour cells *in vitro* and shrink tumors *in vivo*, even in the absence of CAR T cells. While both engineered EVs exhibit potent baseline cytotoxicity, the integration of HPSE transforms HPSE CAR T EVs into a superior standalone effector platform. Beyond passive diffusion, EV‐anchored HPSE enables active matrix remodelling (Figure 4I), which alleviates the physical constraints of the TME. This dual‐action capability—simultaneously dismantling the stromal barrier and delivering enriched death ligands (Figure S10E)—allows HPSE CAR T EVs to achieve more thorough tumour neutralization, leveraging a specialized enzymatic function that is not present in standard CAR T platforms. In addition to high CAR expression, HPSE CAR T EVs also display elevated levels of CCR5, CCR7, FasL, and TRAIL, which promote their accumulation within tumour sites (Gonzalez‐Martin et al., 2011, Roberts et al., 2016, Reichmann, 2002, Daniels et al., 2005). One mechanism is death ligand‐mediated apoptosis (Kornepati et al., 2022). TRAIL rose on treated T cells and on EVs, and DR5 is present on many tumour cells. TRAIL engages DR5 and starts a caspase cascade that drives apoptosis in tumour cells (Deng and Shah, 2020, Guo et al., 2025). Clinical and preclinical work supports TRAIL‐DR5 as a route to kill cancer cells while sparing most normal cells (Amarante‐Mendes and Griffith, 2015)^,^ (Yuan et al., 2018). Importantly, TRAIL can be delivered on the surface of vesicles, and EVs bearing TRAIL have been shown to induce tumour cell death in model systems (Rivoltini et al., 2016). This makes vesicle‐mediated TRAIL delivery a plausible contributor to the tumour killing we see.

Furthermore, the high surface expression of PD‐1 on these EVs allows them to competitively bind PD‐L1 on tumour cells, thereby shielding endogenous T cells from PD‐L1–mediated inhibition and mitigating T cell exhaustion (Chen et al., 2016). Thus, HPSE CAR T EVs emerge as a highly potent, cell‐free nanotherapeutic that can be manufactured, stored, and delivered independently of live cells, harnessing CAR T efficacy with improved safety and flexibility.

Beyond direct cytotoxicity, HPSE CAR T EVs appear to locally modulate the phenotype of adoptively transferred human T cells within the TME. This mirrors observations in other cytotoxic‐lymphocyte EVs, where TRAIL, and costimulatory molecules co‐localize (Lyu et al., 2024). We observed that treatment with these EVs not only induces apoptosis in tumour cells, but also enhances bystander T cell activation and migration. Crucially, while surface ligands initiate contact‐dependent events, the highly efficient cellular internalization of these EVs identifies them as effective intracellular cargo delivery vehicles. This robust uptake provides the physical basis for subsequent deep T‐cell functional modulation and synergistic cytotoxicity. This is consistent with reports that T cell EV contents can upregulate endothelial adhesion receptors and integrins, thus promoting lymphocyte homing (Park et al., 2019). In sum, HPSE CAR T EVs act as immune adjuvants: they bridge innate and adaptive arms by recruiting T cells (chemotaxis) and boosting their function, thereby indirectly eradicating tumour cells that lack the CAR target.

While our NSG‐based model with adoptive transfer of bystander human T cells enables interrogation of adaptive human T cell interactions *in vivo*, we recognize that it does not fully recapitulate the complexity of a complete human immune system, particularly with respect to innate immune populations such as macrophages and NK cells. Consequently, our references to ‘immune modulation’ or ‘remodelling’ specifically reflect the localized phenotypic skewing and functional enhancement of the adoptively transferred human bystander T cells, rather than global systemic immunity. Fully humanized mouse models (e.g., HSC‐engrafted) would be valuable for assessing broader human immune crosstalk. Additionally, orthotopic syngeneic models in immunocompetent mice, such as MC38 colorectal tumors, would provide a more physiologically relevant setting to evaluate EV‐mediated effects on endogenous host immunity and ECM remodelling in a native TME, including metastatic potential. These complementary model systems represent important future directions to build upon the foundational insights gained from the current study.

Taken together, HPSE CAR T cells and their EVs form a synergistic two‐pronged therapy against solid tumors. The HPSE CAR T cells themselves deliver potent on‐target killing and locally digest the fibrotic matrix, carving paths into the tumour. Concurrently, the EVs disseminate antitumor payloads systemically, penetrating regions inaccessible to cells and marshalling the host immune system. This partnership directly addresses two critical obstacles of solid tumors. First, the dense ECM and immunosuppressive stroma that hinder conventional CAR T infiltration are actively broken down by HPSE, overcoming the physical blockade (Li et al., 2018). Second, the problem of antigen heterogeneity—where elimination of target‐positive cells can leave antigen‐negative clones in the shadow is offset by the EV‐mediated immune orchestration. Endogenous cytotoxic T cells, drawn in by EV signals, can seek out and kill those residual antigen‐null cells. Indeed, combining cell‐based and vesicle‐based platforms has been argued to be more effective than either alone. Our strategy exemplifies this principle: CAR T cells provide macroscopic tumour debulking, while CAR T EVs deliver deep stromal penetration and localized cytotoxic effects, together efficiently clearing heterogeneous cancer cell populations. Both the cell and EV products carry the same CAR payload but exhibit different pharmacokinetic profiles, offering combinatorial dosing strategies. In summary, our findings highlight next‐generation immunotherapies that overcome the fibrotic, heterogeneous nature of solid tumors. By engineering CAR T cells with ECM‐degrading power and EV delivery, we create two independent yet compatible agents. Each is capable of significant antitumor activity on its own, but together they embody a comprehensive assault on the TME. These innovations reinforce the promise of dual cell‐EV platforms in oncology, paving the way for safer, more effective treatments of stroma‐rich, antigen‐dispersed malignancies.

## Author Contributions

Conceptualization: Songshan Zhu, Jun Yin, Guangxian Xu. Methodology: Songshan Zhu, Jun Yin, Weiqiang Yang, Yiwei Zeng, Na Huang, Ping Ouyang, Kaisong Huang, Xiaolei Zhao, Rui Chen, Xin Fu, Dan Jiang, Guangxian Xu. Validation: Songshan Zhu, Weiqiang Yang, Yiwei Zeng, Ling Zhang, Cong Yu. Formal analysis: Songshan Zhu, Jun Yin, Dan Jiang, Guangxian Xu. Investigation: Songshan Zhu, Weiqiang Yang, Yiwei Zeng, Ling Zhang, Cong Yu. Resources: Songshan Zhu, Na Huang, Rui Chen, Xin Fu, Guangxian Xu. Writing – Original draft: Songshan Zhu, Guangxian Xu. Writing – Review and editing: all authors. Visualization: Songshan Zhu, Jun Yin, Dan Jiang, Guangxian Xu. Supervision: Songshan Zhu, Dan Jiang, Guangxian Xu. Project administration: Songshan Zhu, Dan Jiang, Guangxian Xu. Funding acquisition: Guangxian Xu.

## Conflicts of Interest

Songshan Zhu, Guangxian Xu are named as inventors on a patent related to HPSE‐pX‐Δ1‐30 CAR T cells therapy. The authors have no other competing interests to declare.

## Resource Availability

Further information and requests for resources and reagents should be directed to and will be fulfilled by the lead contact, Guangxian Xu (xuguangxian@gdmu.edu.cn).

## Materials Availability

All materials used in this study are available upon request to the lead contact.

## Supporting information




**Supporting Information**: jev270310‐sup‐0001‐Figure S1.jpg

**Supporting Information**: jev270310‐sup‐0002‐Figure S2.jpg

**Supporting Information**: jev270310‐sup‐0003‐Figure S3.jpg

**Supporting Information**: jev270310‐sup‐0004‐Figure S4.jpg

**Supporting Information**: jev270310‐sup‐0005‐Figure S5.jpg

**Supporting Information**: jev270310‐sup‐0006‐Figure S6.jpg

**Supporting Information**: jev270310‐sup‐0007‐Figure S7.jpg

**Supporting Information**: jev270310‐sup‐0008‐Figure S8.jpg

**Supporting Information**: jev270310‐sup‐0009‐Figure S9.jpg

**Supporting Information**: jev270310‐sup‐0010‐Figure S10.jpg

**Supporting Information**: jev270310‐sup‐0011‐Figure S11.jpg

**Supporting Information**: jev270310‐sup‐0012‐Figure S12.jpg

**Supporting Information**: jev270310‐sup‐0013‐Figure S13.jpg

## Data Availability

All data generated in this study are available upon request to the lead contact.
